# Crimson Tears: A Rare Case of Hematohidrosis

**DOI:** 10.7759/cureus.68233

**Published:** 2024-08-30

**Authors:** Sreeja Cherukuru, Shivarama Krishna Uppara, Rutvik Raval, Mamatha Chappidi, Dileep Satya K

**Affiliations:** 1 Psychiatry, Sri Venkateswara Medical College, Tirupati, IND; 2 Pediatrics, Sri Venkateswara Medical College, Tirupati, IND; 3 Internal Medicine, B. J. Medical College, Ahmedabad, IND; 4 Internal Medicine, Sri Venkateswara Medical College, Tirupati, IND

**Keywords:** multidisciplinary team, pediatric hematology, eccrine sweat glands, stress-induced, bleeding episodes, pediatric psychiatry, hematohidrosis

## Abstract

Hematohidrosis is a rare clinical disorder characterized by oozing blood from intact skin and mucous membranes in the absence of a bleeding disorder. Most of the cases reported are from Asia. Although etiopathogenesis is unclear, it has been strongly linked to psychological stress. A nine-year-old girl was brought to the hospital with multiple episodes of painless bleeding from her nose and mouth for four days and eyes for three days, lasting four to five minutes each. Her symptoms and a thorough but unrevealing workup, including brain imaging, led to a clinical diagnosis of hematohidrosis, with parental disharmony as the underlying stress factor. Family therapy was recommended, and parent management training regarding the positive and negative reinforcement techniques was given. A significant improvement was observed at her one-month follow-up. This case adds to the current limited literature on hematohidrosis, highlighting its association with psychological stress and the importance of a multidisciplinary approach to management. Future research is warranted to elucidate molecular pathways involved in stress-induced vascular dysfunction and explore targeted therapeutic interventions.

## Introduction

Hematohidrosis is a rare clinical disorder characterized by oozing blood from intact skin and mucous membranes in the absence of a bleeding disorder. The common features of this disorder are bloody sweat and bleeding from various sites like the eyes, scalp, and nose, which is mostly painless, lasting from minutes to hours with spontaneous resolution, and frequently associated with underlying psychological stress [1,2]. Most cases occur in individuals 18 years old or younger, with no predisposition to any gender [3]. Most of the reported cases are from Asia [4]. The earliest mention of hematohidrosis dates back to 15th-century religious art, which depicts Jesus Christ sweating drops of blood from his forehead in the Garden of Gethsemane before the crucifixion [5].

The exact etiology of hematohidrosis is not well understood, but several hypotheses and potential contributing factors have been proposed. The most frequently proposed adrenergic-related mechanism involves constriction of arterioles in the dermal plexus around the eccrine sweat glands during stressful periods, followed by dilation to the point of rupture [6]. Although sympathetic activation due to psychological stress like acute fear and intense mental contemplation is regarded as the most common underlying trigger, physical stress like strenuous exercise or prolonged exposure to heat can also trigger hematohidrosis [6-8].

Hematohidrosis is extremely rare, has no established diagnostic criteria, and remains a diagnosis of exclusion. Differentiating from conditions like coagulopathies, vasculitides, skin conditions like purpura or petechiae, or even self-inflicted injury could be challenging due to the myriad of tests that would be required [7]. The existing literature on hematohidrosis is mainly composed of case reports. Propranolol, by reducing adrenergic activity, can prevent or reduce the occurrence of bleeding episodes by stabilizing blood vessels and decreasing the likelihood of rupture and has been widely used in the management of hematohidrosis [3,9,10,11]. Patients also improved with drugs like atropine sulfate [12] and oxybutinin [13]. Spontaneous resolution can also occur [8]. Since this condition is manageable, early recognition and treatment will immensely benefit the patients [3,9-13]. We hereby report a curious case of hematohidrosis induced by stress in a nine-year-old previously healthy female child.

## Case presentation

**Figure 1:**
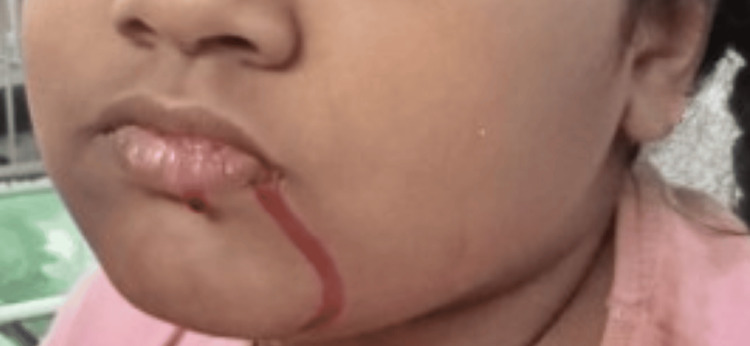
Photograph of the patient showing bleeding from the corner of her mouth

**Figure 2:**
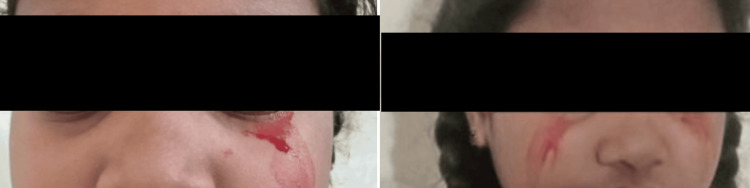
Photographs of the patient showing bleeding from the eyes 2A: bleeding from the left eye, 2B: bleeding from both eyes

A nine-year-old girl was brought to the department of pediatrics of a tertiary care hospital in South India with complaints of bleeding from her nose and mouth (Figure 1) for the past four days and bleeding from her eyes (Figure 2) for the past three days. These episodes lasted for four to five minutes, multiple times of the day, and were not associated with pain.

She had a history of bleeding per vaginum two months ago, which was minimal, just staining the pad, and lasted for four days. There was no history of trauma to the face or genitals, bleeding disorders, psychiatric disorders, recent fevers, abdominal pain, allergy to food or drugs, or excessive consumption of a colored diet. There is no history of psychiatric disorders and bleeding disorders in the family. There were no complaints regarding the child's educational performance.

On further questioning, it was found that the parents were in marital disharmony. The father and mother of the child separated two years ago. The child expressed concerns about being separated from her parents and being alone. She also complained of occasional nightmares, at least once every two weeks.

On examination, she was alert and cooperative. Her intelligence, as observed clinically, is normal. Mild pallor was observed in her lower palpebral conjunctiva. Her BMI was 30.2 kg/m², indicating obesity. During physical examination, no abnormal findings were detected. The secretions were watery and bright red in color. No recurrence was observed once the secretions were wiped off. There were no signs of trauma and skin or mucosal damage in and around the mouth, nose, and eyes, and there was no sign of hair plucking.

As shown in Table 1, no abnormalities were detected in this patient's lab results, with all parameters falling within normal reference ranges. Microscopic examination of the secretions revealed red blood cells. The serum antinuclear antibody (ANA) titer was negative (1:40), and a CT scan of the brain and sinuses, done to rule out tumors, vascular malformations, infections, or any other potential sources of bleeding like hematomas and aneurysms, was unremarkable.

**Table 1:** Laboratory data overview This table illustrates the summary of the laboratory results of the patient. INR: international normalized ratio, APTT: activated partial thromboplastin time, T4: thyroxine, TSH: thyroid-stimulating hormone

Parameters	Patient values	Reference range
Hemoglobin	12 g/dL	11.5-15.5 g/dL
Total leucocyte count	7800 cells/mm^3^	4500-11000 cells/mm^3^
Differential counts (%)		
Neutrophils	60	25-60
Lymphocytes	34	30-60
Monocytes	4	2-10
Eosinophils	1	1-4
Basophils	1	0-1
Platelets	3,96,000 cells/mm^3^	1,50,000-4,00,000 cells/mm^3^
Bleeding time	2.1 minutes	2-8 minutes
Prothrombin time	11.26 seconds	11-14 seconds
INR	1	0.9-1.1
APTT	28.50 seconds	25-40 seconds
D-dimer	0.26 μg/mL	<0.4 μg/mL
Fibrinogen	3.50 g/L	1.69-3.60 g/L
Thyroid function tests		
Free T4	1.0 ng/dL	0.96-1.60 ng/dL
TSH	1.2 mIU/L	0.7-6.4 mIU/L

The patient’s symptoms and a thorough but unrevealing workup led to a clinical diagnosis of hematohidrosis. Family therapy was recommended, and parent management training regarding the positive and negative reinforcement techniques was given. The child received instructions on relaxation techniques and was prescribed propranolol, 20 mg, twice daily, and clonazepam, 0.5 mg, daily. At her one-month follow-up, there was a significant improvement in her symptoms, with only one episode of bleeding from her nose during the first week after starting the treatment. She had no recurrence after that episode. The medications were discontinued, and a re-evaluation after two weeks was recommended.

## Discussion

Comparing the presented case with previous studies, similarities in clinical presentation and response to treatment are evident. Like other reported cases, this patient exhibited bleeding from multiple sites without evidence of trauma or underlying pathology [1,3,6,8-10,12-14]. The response to family therapy and pharmacotherapy aligns with existing literature, emphasizing the importance of addressing underlying psychological stressors in managing hematohidrosis [10]. However, it is noteworthy that the patient's symptoms resolved with a relatively low dose of propranolol and clonazepam, suggesting the need for further investigation into optimal pharmacological management strategies.

Hematohidrosis can mimic various conditions that could cause cutaneous bleeding. Bleeding disorders like hemophilia, von Willebrand disease, platelet function disorders, and vasculitides like Henoch-Schönlein purpura or systemic lupus erythematosus can present with cutaneous bleeding [15]. Self-inflicted injuries or malingering should also be ruled out, especially in the absence of a clear medical cause [16]. Strengths of this study include a comprehensive evaluation ruling out underlying bleeding disorders and vasculitides and a multidisciplinary approach to management involving family therapy and psychotropic medications. However, limitations include the lack of long-term follow-up data and the absence of objective measures to assess stress levels and treatment response. Additionally, the precise mechanisms underlying stress-induced hematohidrosis remain speculative, highlighting the need for further research into the pathophysiology of this condition [9,11,12].

The hypothesis that stress-induced dysregulation of the sympathetic nervous system contributes to hematohidrosis underscores the significance of addressing psychological well-being in clinical practice [1,10,13]. Future research should aim to elucidate the molecular pathways involved in stress-induced vascular dysfunction and explore targeted therapeutic interventions. Longitudinal studies assessing the long-term outcomes of patients with hematohidrosis and evaluating the efficacy of different treatment modalities are warranted to optimize management strategies and improve patient outcomes. Furthermore, to avoid complications like psychological distress, social and functional impairment, and anemia, continued awareness and education among healthcare professionals regarding this rare condition are crucial for timely recognition and appropriate management [7,10,13].

## Conclusions

The presented case highlights the rarity of hematohidrosis and its potential association with psychological stress, particularly in children. The manifestation of bleeding from multiple sites, including the nose, mouth, and eyes, coupled with a history of bleeding per vaginum underscores the diverse presentation of this condition. The absence of any underlying bleeding disorder or significant abnormalities in coagulation parameters suggests a primary etiology related to stress-induced vascular dysfunction. The prompt improvement of symptoms with family therapy and pharmacological interventions further supports the hypothesis of stress as a precipitating factor in hematohidrosis.

The presented case adds to the limited literature on hematohidrosis, emphasizing its association with psychological stress and the importance of a multidisciplinary approach to management. The successful management of family therapy and pharmacological interventions supports the hypothesis of stress as a precipitating factor in hematohidrosis. Further research is needed to better understand the underlying mechanisms and optimize treatment strategies for this enigmatic condition.
